# Supplementary figures and images for: Long-term benefit of vasodilating beta-blockers in acute myocardial infarction patients with mildly reduced left ventricular ejection fraction
Source: PLoS One. 2025 Jun 23;20(6):e0326516. doi: 10.1371/journal.pone.0326516 (PMC12184898; doi:10.1371/journal.pone.0326516)

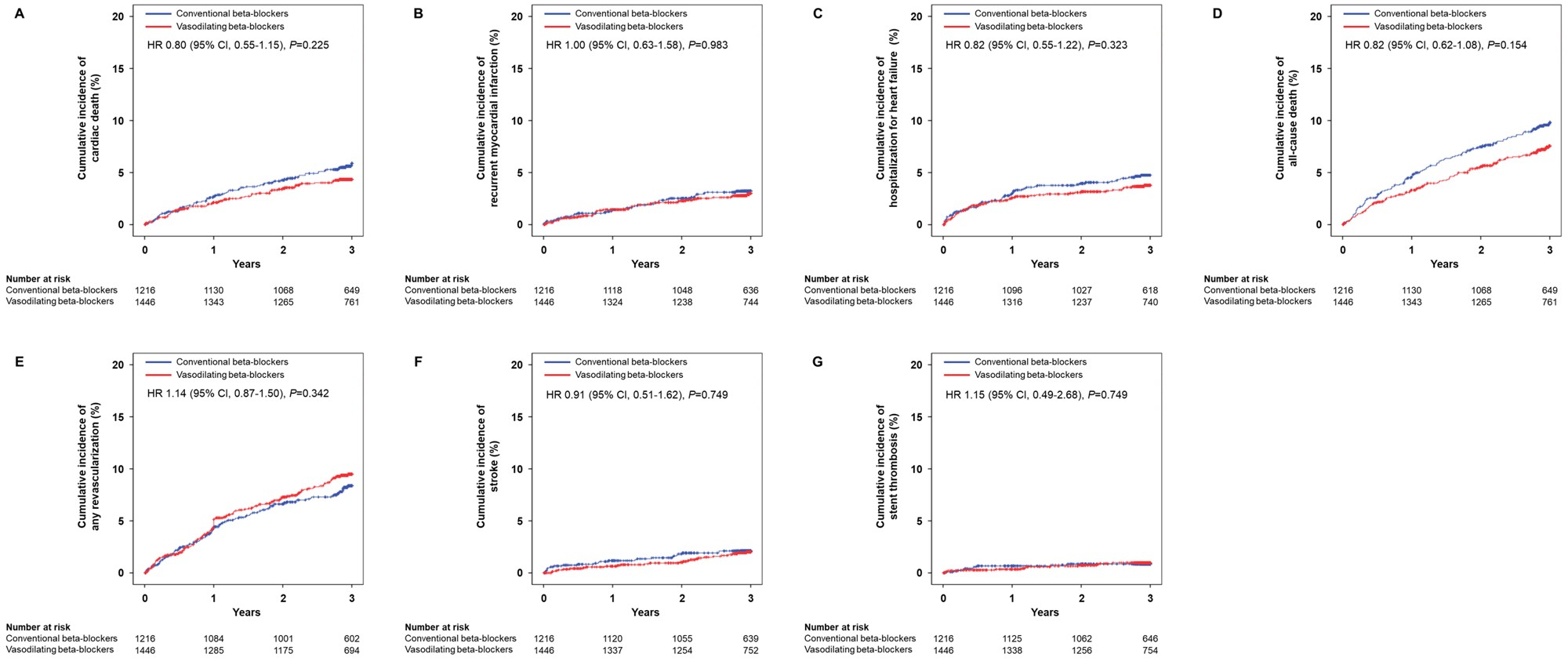

Supplement: S1 Fig — (A) Cardiac death (B) Recurrent myocardial infarction. (C) Hospitalization for heart failure. (D) All-cause death. (E) Any revascularization. (F) Stroke. (G) Stent thrombosis. CI, confidence interval. (TIF) [file pone.0326516.s001.tif]

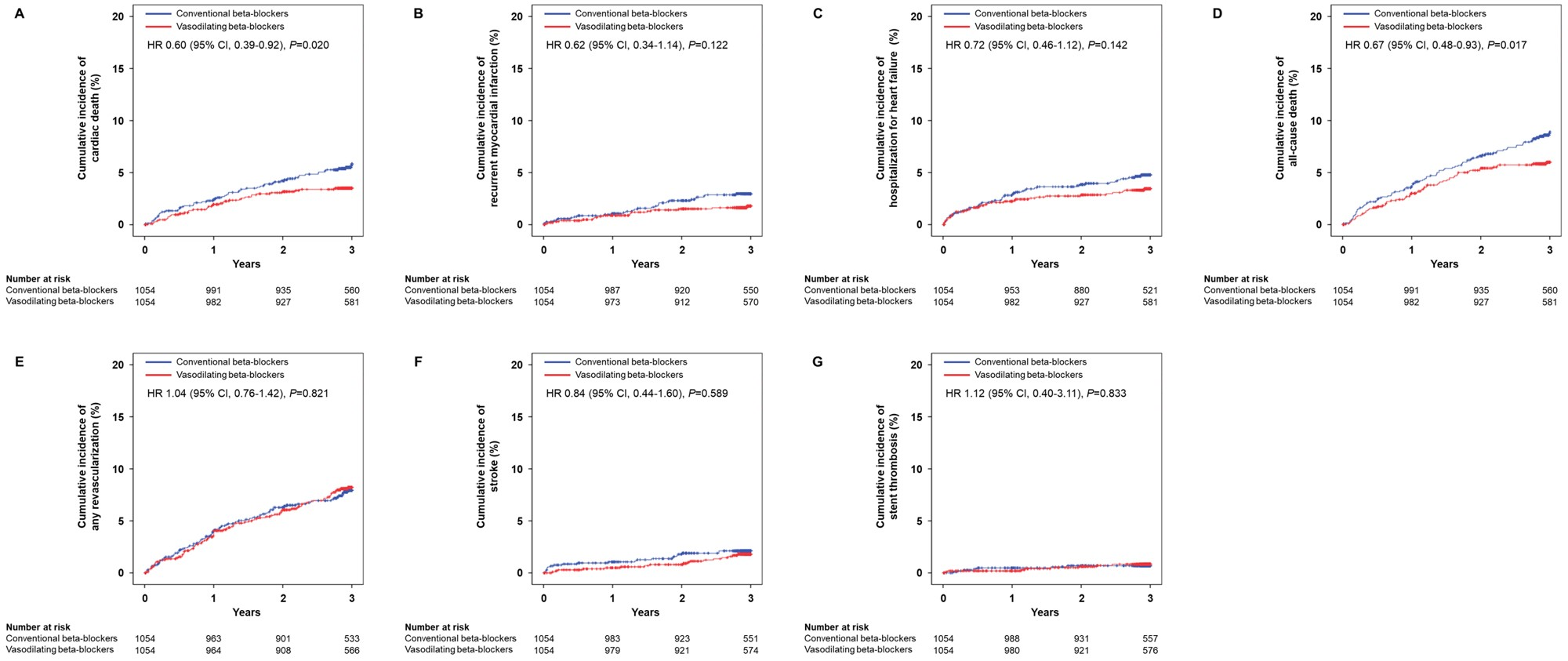

Supplement: S2 Fig — (A) Cardiac death (B) Recurrent myocardial infarction. (C) Hospitalization for heart failure. (D) All-cause death. (E) Any revascularization. (F) Stroke. (G) Stent thrombosis. CI, confidence interval. (TIF) [file pone.0326516.s002.tif]
