# Supplementary material for: Long-term benefit of vasodilating beta-blockers in acute myocardial infarction patients with mildly reduced left ventricular ejection fraction
Source: PLoS One. 2025 Jun 23;20(6):e0326516. doi: 10.1371/journal.pone.0326516 (PMC12184898; doi:10.1371/journal.pone.0326516)
Supplement: S1 Table — (PDF) [file pone.0326516.s003.pdf]

S1 Table. Reperfusion rates and methods in the entire cohort

	All patients (n=2,662)	Vasodilating beta-blockers (n=1,446)	Conventional beta-blockers (n=1,216)
Coronary reperfusion			
Yes[a]	2,587 (97.2)	1,409 (97.4)	1,178 (96.9)
No	75 (2.8)	37 (2.6)	38 (3.1)
Percutaneous coronary intervention	2,517 (94.6)	1,376 (95.2)[b]	1,141 (93.8)
Stent	2,379 (89.4)	1,295 (89.6)	1,084 (89.1)
Drug-eluting stent	2,319 (87.1)	1,279 (88.5)	1,040 (85.5)
Bare metal stent	60 (2.3)	16 (1.1)	44 (3.6)
Balloon only	133 (5.0)	76 (5.3)	57 (4.7)
Thrombus aspiration	5 (0.2)	5 (0.3)	0 (0.0)
Thrombolysis	5 (0.2)	1 (0.1)	4 (0.3)
Coronary artery bypass graft	32 (1.2)	21 (1.5)	11 (0.9)
None			
MINOCA	39 (1.5)	14 (1.0)	25 (2.1)
Insignificant lesion	38 (1.4)	14 (1.0)	24 (2.0)
Coronary artery spasm	1 (0.0)	0 (0.0)	1 (0.1)
Myocardial bridge	1 (0.0)	0 (0.0)	1 (0.1)
Conservative management	68 (2.6)	34 (2.4)	34 (2.8)

Values are number (%).

MINOCA, myocardial infarction with non-obstructed coronary arteries

[a]Included MINOCA and myocardial bridge.

[b]*P*<0.05 vs. with Conventional beta-blockers
