# Supplementary material for: Long-term benefit of vasodilating beta-blockers in acute myocardial infarction patients with mildly reduced left ventricular ejection fraction
Source: PLoS One. 2025 Jun 23;20(6):e0326516. doi: 10.1371/journal.pone.0326516 (PMC12184898; doi:10.1371/journal.pone.0326516)
Supplement: S2 Table — (PDF) [file pone.0326516.s004.pdf]

S2 Table. Reperfusion rates and methods in propensity-score matched cohort

	All patients (n=2,108)	Vasodilating beta-blockers (n=1,054)	Conventional beta-blockers (n=1,054)
Coronary reperfusion			
Yes[a]	2,053 (97.4)	1,027 (97.4)	1,026 (97.3)
No	55 (2.6)	27 (2.6)	28 (2.7)
Percutaneous coronary intervention	2,003 (95.0)	1,007 (95.5)	996 (94.5)
Stent	1,902 (90.2)	954 (90.5)	948 (89.9)
Drug-eluting stent	1,851 (87.8)	942 (88.5)	909 (86.2.)
Bare metal stent	51 (2.4)	12 (1.1)	39 (3.7)
Balloon only	97 (4.6)	49 (4.6)	48 (4.7)
Thrombus aspiration	4 (0.2)	4 (0.4)	0 (0.0)
Thrombolysis	4 (0.2)	0 (0.0)	4 (0.4)
Coronary artery bypass graft	23 (1.1)	12 (1.1)	11 (1.0)
None			
MINOCA	27 (1.5)	9 (0.9)	18 (2.1)
Insignificant lesion	25 (1.2)	9 (0.9)	16 (1.5)
Coronary artery spasm	1 (0.0)	0 (0.0)	1 (0.1)
Myocardial bridge	1 (0.0)	0 (0.0)	1 (0.1)
Conservative management	51 (2.4)	26 (2.5)	25 (2.4)

Values are number (%).

MINOCA, myocardial infarction with non-obstructed coronary arteries

[a]Included MINOCA and myocardial bridge.
