# Supplementary material for: Long-term benefit of vasodilating beta-blockers in acute myocardial infarction patients with mildly reduced left ventricular ejection fraction
Source: PLoS One. 2025 Jun 23;20(6):e0326516. doi: 10.1371/journal.pone.0326516 (PMC12184898; doi:10.1371/journal.pone.0326516)
Supplement: S3 Table — (PDF) [file pone.0326516.s005.pdf]

S3 Table. Generic names and doses of beta-blockers prescribed at discharge in propensity-score matched cohort.

Generic name	No. of patients	Mean dose (mg)	Median dose (mg)
Vasodilating beta-blockers (n=1,054)			
Carvedilol	946 (89.8)	6.25±6.7	6.25 (3.125-6.25)
Nebivolol	108 (10.2)	2.8±1.5	2.5 (1.25-5.0)
Conventional beta-blockers (n=1,054)			
Bisoprolol	1022 (97.0)	1.9±1.2	1.25 (1.25-2.50)
Metoprolol	32 (3.0)	58.6±20.7	50 (50-50)

Values are number (%), mean±standard deviation, or median (interquartile range).
