# Supplementary material for: Long-term benefit of vasodilating beta-blockers in acute myocardial infarction patients with mildly reduced left ventricular ejection fraction
Source: PLoS One. 2025 Jun 23;20(6):e0326516. doi: 10.1371/journal.pone.0326516 (PMC12184898; doi:10.1371/journal.pone.0326516)
Supplement: Attachment — Submitted filename: Response to reviewers-0503.docx [file pone.0326516.s007.docx]

**Reviewer #1**

1. Residual Confounding

While PSM is a valid approach, unmeasured confounders (e.g., medication adherence, renal function, physician preference) may still exist.

Suggestion: The authors may consider acknowledging this as a limitation in the discussion.

- Thank you very much for bringing this to our attention.
- We would like to clarify that medication adherence was already acknowledged as a limitation in the original manuscript; however, we have revised and clarified this point further in the revised version. Additionally, we have included a new statement addressing the limitation related to renal function, emphasizing that it was classified by CKD stage rather than estimated GFR, which may have limited the precision of renal risk stratification. Regarding physician treatment preference, this was already acknowledged as a limitation in the initial version, and thus, no additional changes were made in this respect.
- We added the following to the limitation in the discussion section:

Although PSM was employed to adjust for baseline differences, unmeasured and residual confounding factors, as well as possible selection bias, may have persisted. Notably, renal function was stratified using CKD stages rather than estimated glomerular filtration rate, which may have limited the granularity of renal risk assessment. Treatment selection between vasodilating and conventional beta-blockers after PCI was also based on physician preference, which may have introduced allocation bias beyond the control of statistical matching.

2. Vasodilating Beta-Blocker Subgroup Analysis

The study groups all vasodilating beta-blockers (Carvedilol, Nebivolol) together despite different mechanisms. However, since the authors report no significant difference between these agents, separate subgroup analysis is not necessary.

Suggestion: Briefly discuss the availability of Nebivolol in post-MI treatment guidelines (as some countries prefer Carvedilol due to stronger RCT evidence).

- Thank you for your comment.
- We analyzed clinical outcomes by dividing patients into carvedilol group and nebivolol group, and found no significant differences between the two groups in terms of primary outcome (7.7% vs 6.5%, HR 1.29, 95% CI: 0.58-2.86, *P*=0.531), cardiac death (3.3% vs 3.7%, HR 0.73, 95% CI: 0.25-2.14, *P*=0.568 ), MI (1.5% vs 2.8%, HR 0.82, 95% CI: 0.22-3.01, *P*=0.759), hospitalization due to HF (3.6% vs 0.9%, HR 6.05, 95% CI: 0.83-44.14, *P*=0.076), all-cause death (5.6% vs 6.5%, HR 0.76, 95% CI: 0.34-1.70, *P*=0.500), any revascularization (7.6% vs 6.5%, HR 1.43, 95% CI: 0.65-3.16, *P*=0.376), stroke (1.5% vs 2.8%, HR 0.52, 95% CI: 0.15-1.84, *P*=0.307), and stent thrombosis (0.8% vs 1.8%, HR 0.47, 95% CI: 0.23-1.76, *P*=0.106).
- We added the following to the Discussion section:

Although limited evidence exists regarding the efficacy of nebivolol in post-MI patients, prior studies reported that nebivolol was associated with fewer cardiovascular events compared to metoprolol or conventional beta-blockers, with outcomes comparable to those observed in the carvedilol group. (26,27)

Reference)

26.	Ozaydin M, Yucel H, Kocyigit S, Adali MK, Aksoy F, Kahraman F, et al. Nebivolol versus Carvedilol or Metoprolol in Patients Presenting with Acute Myocardial Infarction Complicated by Left Ventricular Dysfunction. Med Princ Pract. 2016;25(4):316-22.

27.	Chung J, Han JK, Yang HM, Park KW, Kang HJ, Koo BK, et al. Long-term efficacy of vasodilating beta-blocker in patients with acute myocardial infarction: nationwide multicenter prospective registry. Korean J Intern Med. 2021;36(Suppl 1):S62-S71.

3. Medication Adherence Dose Titration

Was medication adherence assessed? If patients on vasodilating beta-blockers were more adherent, this could bias results.

Suggestion: Acknowledge this limitation in the discussion.

- We appreciate your valuable comment.
- We assessed the adherence to medication. Medication adherence was indirectly assessed based on prescription data at follow-up. In the entire cohort, 97% of patients discharged on vasodilating beta-blockers remained on the same regimen at 1 year, compared to 95% in patients with conventional beta-blockers. At 2 years, persistence rates were identical between the groups, with 73% of patients with both the vasodilating and conventional beta-blockers continuing their initially prescribed therapy. In the PSM cohort, medication persistence showed a comparable trend: 97% and 74% of patients with vasodilating beta-blockers remained on therapy at 1 and 2 years, respectively, compared with 96% and 74% of patients with conventional beta-blockers. There are no significant differences between vasodilating beta-blockers and conventional beta-blockers.
- We added the following to the limitation in the discussion section:

Additionally, the study did not capture detailed information on beta-blocker dosing, therapy modifications during follow-up, adverse events, or direct measures of medication adherence. Adherence was inferred from prescription continuity at follow-up visits and appeared similar between treatment groups over 1- and 2-year intervals; however, this indirect measure does not confirm actual medication intake. Furthermore, real-time adherence behavior, dose titrations, and interim changes in therapy were not systematically recorded.

Reviewer #2:

1. The authors need to specify how was LVEF obtained? Echo only? CMR?, do they have inter or intravariability analysis? Did LVEF was assessed in some core lab? This is relevant since the range between 41-49% is small and subjected to variability. This is also a problem with the current HFmrEF classification for patients with HF.

- Thank you for your comment.
- We added the following to the limitation in the discussion section:

LVEF was determined through echocardiography, with mrEF classification based exclusively on these imaging results. Echocardiographic assessments were conducted independently at each participating institution according to routine clinical protocols, without subsequent adjudication by a centralized core laboratory. Consequently, potential variability in measurement across centers may have affected the precision of LVEF categorization, particularly in cases near the mrEF threshold, where minor deviations could influence group assignment.

2. Do they have any data on the use of other medications? SGLT2i, MRA, ARNI?

- Thank you very much for bringing this to our attention.
- The KAMIR-NIH registry does not include detailed information on the use of certain heart failure-specific medications such as SGLT2 inhibitors, MRAs, or ARNIs. As the registry was primarily designed to investigate acute myocardial infarction rather than heart failure management, comprehensive data on all heart failure therapies were not systematically collected. Furthermore, the enrollment period of the registry (2011–2015) preceded the widespread adoption of SGLT2 inhibitors and ARNIs in Korean clinical practice, which may further explain the absence of these variables.
- We added the following to the limitation in the discussion section:

The registry also did not systematically record the use of important heart failure-related medications such as sodium glucose cotransporter 2 inhibitor, mineralocorticoid receptor antagonists and angiotensin receptor neprilysin inhibitor. This limitation reflects the AMI-focused scope of the KAMIR-NIH registry, which was not specifically designed to capture the full spectrum of heart failure pharmacotherapy. These gaps raise the possibility of therapeutic confounding over the course of follow-up.

3. Do they have data on LVEF recovery? How many patients developed heart failure? And how many showed a recovery? Is there any difference between vasodilating and non-vasodilating betablockers?

- We appreciate the reviewer’s interest in the longitudinal changes in LVEF.
- Follow-up echocardiography was not universally performed, as it was not mandated by the study protocol and was conducted at the discretion of the treating physician. In the entire cohort, follow-up echocardiography was available in 41% of patients at 1 year, 19% at 2 years, and 18% at 3 years. Among these, the proportions of patients showing a ≥10% improvement in LVEF were slightly higher in the vasodilating beta-blocker group compared to the conventional group, but these differences were not statistically significant at any time point. Specifically, at 1 year, the rate of significant LVEF improvement (≥10%) was 13.4% in the vasodilating beta-blocker group and 11.6% in the conventional group; at 2 years, 7.8% vs. 7.2%; and at 3 years, 5.9% vs. 5.6%, respectively. A similar trend was observed in the PSM cohort, with 14.2% vs. 13.2% at 1 year, 6.3% vs. 5.8% at 2 years, and 5.8% vs. 5.1% at 3 years for vasodilating and conventional beta-blockers, respectively. The proportions of patients who experienced a decline in EF or minimal changes (0–10%) were also comparable between groups. Overall, there were no statistically significant differences in LVEF recovery between vasodilating and conventional beta-blockers in either the entire or PSM cohorts.

4. The authors state that there is a reduction of heart failure hospitalizations, but this seems to be non-significant.

- Thank you very much for your comments.
- We agree with the reviewer’s comments. Although the incidence of hospitalization for heart failure was numerically lower in the vasodilating beta-blocker group, the difference did not reach statistical significance in our analysis. This has been presented in Table 2 as the results of both the entire cohort and the PSM cohort.

5. The reduction in the primary outcome seems to be driven by Cardiac death and all cause death but no HFH, so there should be another explanation for the possible benefit different from afterload reduction and remodeling…

- Thank you for the great comments.
- We added the following in the Discussion section:
- While the rates of hospitalization due to heart failure were comparable between vasodilating beta-blockers and conventional beta-blockers, the lower cardiac and all-cause mortality observed with vasodilating beta-blockers suggests potential benefits extending beyond conventional hemodynamic mechanisms such as afterload reduction or ventricular remodeling. Vasodilating beta-blockers exhibit distinct pharmacologic profiles, including antioxidative activity, enhancement of endothelial function, and improved coronary microvascular perfusion. (27, 36, 37) These additional effects, which are not observed with conventional beta-blockers, may contribute to their favorable impact on survival in patients with AMI and offer a plausible explanation for observed difference in the mortality in our analysis.

References)

27 .Chung J, Han JK, Yang HM, Park KW, Kang HJ, Koo BK, et al. Long-term efficacy of vasodilating beta-blocker in patients with acute myocardial infarction: nationwide multicenter prospective registry. Korean J Intern Med. 2021;36(Suppl 1):S62-S71.

36. DiNicolantonio JJ, Lavie CJ, Fares H, Menezes AR, O'Keefe JH. Meta-analysis of carvedilol versus beta 1 selective beta-blockers (atenolol, bisoprolol, metoprolol, and nebivolol). Am J Cardiol. 2013;111(5):765-9.

37. Sorrentino SA, Doerries C, Manes C, Speer T, Dessy C, Lobysheva I, et al. Nebivolol exerts beneficial effects on endothelial function, early endothelial progenitor cells, myocardial neovascularization, and left ventricular dysfunction early after myocardial infarction beyond conventional beta1-blockade. J Am Coll Cardiol. 2011;57(5):601-11.

Reviewer #3:

1. Pharmacotherapy adjustement during follow-up migh be inadequate, we do not know which patients were using MRAs, loop diuretics, etc. and in which doses. I am aware that authors adjusted their analysis for P2Y12 inhibitors, RAAS inhibitors and statins, however we do not know what occured with other relevant medications in this population, although many parameters were taken into account regarding PSM co-variate adjustment.

- Thank you for the great comments. We agree with the reviewer’s valuable comment.
- Unfortunately, information on the use and dosing of certain heart failure-specific medications such as MRAs or loop diuretics was not collected in the KAMIR-NIH registry. This is likely because the registry was primarily designed to evaluate AMI rather than chronic heart failure management. In addition, medication adherence was assessed only at predefined outpatient follow-up visits based on prescription data, and actual medication intake or dose titration could not be monitored.
- We added the following to the limitation in the discussion section:

The registry also did not systematically record the use of important heart failure-related medications such as sodium glucose cotransporter 2 inhibitor, mineralocorticoid receptor antagonists and angiotensin receptor neprilysin inhibitor. This limitation reflects the AMI-focused scope of the KAMIR-NIH registry, which was not specifically designed to capture the full spectrum of heart failure pharmacotherapy. These gaps raise the possibility of therapeutic confounding over the course of follow-up.

2. How do the authors explain that vasodilating BBs had stronger impact in patients with chronic kidney disease versus ones that did not have it? What is the biological rationale and plausibility behind this? Similarly, why did the patients that were older had more benefit from it, compared to younger ones?

- Thank you for the great comments.
- We appreciate the reviewer’s insightful comment. The more pronounced benefit of vasodilating beta-blockers in patients with CKD may be attributable to their favorable hemodynamic and endothelial effects, which are particularly relevant in this population. Vasodilating beta-blockers reduces peripheral vascular resistance and improve endothelial function - mechanisms that may help mitigate the progression of cardiorenal syndrome and reduce cardiovascular stress in patients with impaired renal function. Furthermore, their antioxidative and anti-inflammatory properties may offer additional protective effects in CKD, where oxidative stress and systemic inflammation are commonly elevated.

Similarly, the greater benefit observed in older patients could be explained by the age-related decline in vascular compliance and increased arterial stiffness, both of which may be better addressed by the vasodilatory properties of these agents. In this context, vasodilating beta-blockers may offer more comprehensive cardiovascular protection than conventional beta-blockers, particularly in high-risk populations such as older adults and those with CKD. We have included this possible biological rationale in the revised discussion.

- Subgroup analyses suggested that the mortality benefit associated with vasodilating beta-blockers was more pronounced in patients with CKD and in older individuals. In patients with CKD, vasodilating beta-blockers confer hemodynamic benefits, namely a reduction in systemic vascular resistance and enhancement of endothelial function, which may attenuate the deleterious cardiorenal interactions commonly observed in this population. Furthermore, their pleiotropic properties, including antioxidative and anti-inflammatory effects, may provide additional protection in the context of heightened systemic stress.(38) Among older patients, who are more likely to exhibit increased arterial stiffness and vascular dysfunction, the vasodilatory action of these agents may contribute to improved vascular compliance and perfusion.(39) These factors may partly explain the enhanced clinical benefit observed in these subgroups, compared to younger or non-CKD patients.

References)

38.	Wali RK, Iyengar M, Beck GJ, Chartyan DM, Chonchol M, Lukas MA, et al. Efficacy and safety of carvedilol in treatment of heart failure with chronic kidney disease: a meta-analysis of randomized trials. Circ Heart Fail. 2011;4(1):18-26.

39.	Patel K, Fonarow GC, Ekundayo OJ, Aban IB, Kilgore ML, Love TE, et al. Beta-blockers in older patients with heart failure and preserved ejection fraction: class, dosage, and outcomes. Int J Cardiol. 2014;173(3):393-401.

3. There is also lack of characterizing severity burden of coronary disease which might have significantly impact on the outcomes that authors report. How many had bifurcation lesions and had bifurcation stenting? How many had LM disease? Authors also do not provide any objective parameter for this such as for example SYNTAX score or similar. This is a limitation to this study.

- We thank the reviewer for this insightful observation.
- Due to inherent limitations of the KAMIR-NIH registry, detailed anatomical complexity such as presence of bifurcation lesions or bifurcation stenting was not available, and objective scoring systems like the SYNTAX score could not be calculated for this analysis. However, information regarding LM disease was available: in the entire cohort, 46 patients (1.7%) had LM involvement, and in the PSM cohort, 36 patients (1.7%) had LM disease. We have acknowledged this limitation in the Discussion section.
- Due to registry constraints, detailed coronary anatomical data-such as bifurcation lesions and stenting strategies-were unavailable, and validated angiographic risk scores like the SYNTAX score could not be applied. Although the presence of LM disease was documented and infrequent across both cohorts (1.7%), the absence of detailed anatomical characterization limits our ability to assess lesion complexity as a factor influencing outcomes.

4. Finally, mean doses of adminstered beta-blockers seem rather low in the conventional BB groups. Just a 1.9 mg of bisoprolol on average and 58 mg of metoprolol. Could authors adjust their analysis adjusted for the dosages. We know that this might be a considerable limitations since we know that there is incremental survival and other gains increasing with higher doses of BBs administered, in HF in particular. If these patients were underdosed then we cannot expect beneficial effects of these drugs as tested in pivotal randomized controlled trials. This critical fact needs to be addressed.

- We appreciate the reviewer’s important observation regarding beta-blocker dosing.
- As noted, the average doses of conventional beta-blockers were lower than target doses used in prior randomized trials. However, dose data was recorded at discharge and may not fully reflect up-titration during follow-up, which was not systematically captured in the registry. Furthermore, detailed dose information was not available for all patients, precluding adjustment for dose in the main analysis. We acknowledge that underdosing, especially in patients with HF, may attenuate the potential benefits of conventional beta-blockers. This limitation has been added to the Discussion section of the manuscript.
- Moreover, conventional beta-blockers were generally prescribed at sub-target doses relative to those recommended in heart failure clinical trials. Although this likely reflects real-world prescribing behavior in AMI settings, suboptimal dosing may have attenuated their observed effectiveness. As the dose information was only available at discharge and follow-up titration was not systematically documented, we were unable to incorporate dose intensity into the PSM model. Thus, variation in dosing across groups represents a further source of potential bias.

Reviewer #4:
1) the authors use a propensity matching approach to compare the effect of vasodilating vs conventional beta blocker to assess the effects of different beta blocker treatment on patients with similar clinical characteristics. On page 6 the authors indicate the matching strategy was made using a propensity score with nearest neighbor matching algorithm, matched with a caliper width equal to 0.1 of the SD of the propensity score. It would be useful if the authors could clarify how the propensity score is derived, and what exactly the caliper width refers to.

- We thank the reviewer for the valuable comment. The propensity score was estimated using a multivariable logistic regression model in which treatment group (vasodilating vs. conventional beta-blocker) was the dependent variable, and all baseline clinical variables listed in Table 1 were included as covariates. Nearest neighbor 1:1 matching without replacement was performed, using a caliper width of 0.1 standard deviations of the logit of the propensity score, as recommended by previous methodological guidelines. We have clarified this methodological detail in the revised Methods section.
- Propensity scores were estimated using a multivariable logistic regression model in which treatment group (vasodilating or conventional beta-blocker) was the dependent variable. All baseline variables listed in Table 1 were included as covariates. Patients were then matched 1:1 using nearest neighbor matching without replacement, applying a caliper width of 0.1 standard deviations of the logit of the propensity score. This caliper threshold was chosen to minimize residual bias while preserving sample size, based on established recommendations for optimal matching quality.

2) Is there any relationship in the data between LV EF (40-50%) and event rates? Do vasodilating beta blockers have greater effect in lower LV EF or no clear effect?

- Thank you for the great comments.
- We analyzed the relationship between LVEF and clinical outcomes. In entire cohort, there are no relationships between LVEF and primary outcome (HR 0.97; 95% CI: 0.94-1.02; *P*=0.274), cardiac death (HR 0.99; 95% CI: 0.91-1.07; *P*=0.707), MI (HR 0.96; 95% CI: 0.86-1.07; *P*=0.478), and hospitalization for HF (HR 1.00; 95% CI: 0.92-1.09; *P*=0.955). Also, in PSM cohort, there are no relationships between LVEF and primary outcome (HR 0.98; 95% CI: 0.93-1.04; *P*=0.557), cardiac death (HR 0.95; 95% CI: 0.89-1.02; *P*=0.185), MI (HR 0.96; 95% CI: 0.88-1.01; *P*=0.298), and hospitalization for HF (HR 0.99; 95% CI: 0.94-1.07; *P*=0.773).
- We analyzed the effect of vasodilating beta-blockers according to LVEF.
- In entire cohort, there was no statistically significant interaction between LVEF and beta-blocker type (vasodilating vs. conventional) on the primary outcome (*β*=0.602; 95% CI: -0.006 to 0.011; *P*=0.547), cardiac death (*β*=-0.026; 95% CI: -0.006 to 0.006; *P*=0.934), MI (*β*=-0.321; 95% CI: -0.002 to 0.007; *P*=0.314), and hospitalization for HF (*β*=-0.322; 95% CI: -0.003 to 0.008; *P*=0.312) suggesting that the effect of vasodilating beta-blockers was not clearly modified by baseline LVEF. Also, in PSM cohort, there was no statistically significant interaction between LVEF and beta-blocker type on the primary outcome (*β*=0.787; 95% CI: -0.007 to 0.016; *P*=0.432), cardiac death (*β*=0.219; 95% CI: -0.004 to 0.008; *P*=0.541), MI (*β*=0.587; 95% CI: -0.002 to 0.009; *P*=0.063), and hospitalization for HF (*β*=0.288; 95% CI: -0.004 to 0.008; *P*=0.422). Therefore, vasodilating beta-blockers did not demonstrate a greater effect in patients with lower LVEF compared to those with higher LVEF.
- We decided not to include this analysis in the manuscript, as the interaction term was not statistically significant and did not alter the overall interpretation of the results.
