# Supplementary material for: Long-term benefit of vasodilating beta-blockers in acute myocardial infarction patients with mildly reduced left ventricular ejection fraction
Source: PLoS One. 2025 Jun 23;20(6):e0326516. doi: 10.1371/journal.pone.0326516 (PMC12184898; doi:10.1371/journal.pone.0326516)
Supplement: Attachment — Submitted filename: Minor revision_response to reviewers.docx [file pone.0326516.s008.docx]

Reviewer #1:

The revised manuscript has successfully addressed the previous reviewer comments. The study is technically sound and well-conducted using a large national registry (KAMIR-NIH), and the propensity score-matched analysis is appropriate. The clarification of limitations (e.g., residual confounding, medication adherence, subgroup rationale) enhances the transparency and integrity of the findings. The authors provided clear explanations regarding the observed benefits of vasodilating beta-blockers in subgroups such as CKD and elderly patients. The clinical message is relevant, especially in the context of real-world use of beta-blockers post-AMI in patients with mildly reduced LVEF. I recommend acceptance of this version.

- Thank you for your thorough review and encouraging feedback on our revised manuscript. We are grateful for the constructive suggestions that have helped us improve the clarity and quality of our work.

Reviewer #2:

Thanks to the authors for the great effort addressing our comments.
Please try to include the data used in your responses as a part of the results. Example, you showed data on LVEF longitudinal changes as a part of your responses to the reviewers, but that data is not in the manuscript, and seems to be valuable.

- We sincerely thank you for the valuable comments and kind words. As suggested, we have now incorporated the LVEF longitudinal change data into the Results section of the revised manuscript:

Follow-up echocardiography was available in 41% of patients at 1-year, 19% at 2-year, and 18% at 3- year. Among those with follow-up data, the proportion of patients showing a ≥10% improvement in LVEF at 1 year was slightly higher in the vasodilating beta-blocker group compared to the conventional beta-blocker group (13.4% vs. 11.6%; *P*=0.104 in the entire cohort, 14.2% vs. 13.2%; *P*=0.331 in the PSM cohort), though these differences were not statistically significant. Similar trends were observed at 2-year (7.8% vs. 7.2%; *P*=0.554 in the entire cohort, 6.3% vs. 5.8%; *P*=0.430 in the PSM cohort) and at 3-year (5.9% vs 5.6%; *P*=0.716 in the entire cohort, 5.8% vs. 5.1%; *P*=0.538 in the PSM cohort). The proportions of patients who experienced a decline or minimal changes (0–10%) in EF were also comparable between groups. Overall, there were no statistically significant differences in LVEF recovery between vasodilating and conventional beta-blockers in either the entire or PSM cohorts.

Reviewer #3:

Thank you for answering my queries. No further questions. While the paper still has some limits, most of the important issues have been dealt with.

- We appreciate your thoughtful feedback throughout the review process. We are pleased to hear that the major concerns have been addressed to your satisfaction. Although certain limitations remain, we are grateful for your recognition that the key issues have been adequately dealt with. Thank you for your time and valuable input.

Reviewer #4:

The authors have adequately addressed the queries from reviewers and taken care to modify the text of the article to reflect areas of uncertainty raised by reviewers.

- We sincerely thank you for the constructive comments and positive evaluation. We are glad that the revisions and clarifications made to the manuscript have adequately addressed the concerns raised. Your recognition of our efforts to reflect areas of uncertainty is much appreciated.
